# Antibacterial Drug Development: A New Approach Is Needed for the Field to Survive and Thrive

**DOI:** 10.3390/antibiotics9070412

**Published:** 2020-07-15

**Authors:** M. Courtney Safir, Sujata M. Bhavnani, Christine M. Slover, Paul G. Ambrose, Christopher M. Rubino

**Affiliations:** Institute for Clinical Pharmacodynamics, Inc., Schenectady, NY 12305, USA; csafir@icpd.com (M.C.S.); sbhavnani@icpd.com (S.M.B.); cslover@icpd.com (C.M.S.); pambrose@icpd.com (P.G.A.)

**Keywords:** antibiotic development, monoclonal antibodies, antibiotic marketplace

## Abstract

It is often said that the marketplace for new antibiotics is broken. This notion is supported by the observation that many recently-approved antibiotics to treat drug-resistant bacteria have failed commercially in a spectacular fashion. Today, companies with peak market-cap values in excess of USD 500 million to 1 billion prior to product launch regularly sell for pennies on the dollar a few years after market introduction. It is possible, however, that the market is not as broken as we perceive. That is, in the collective mind of the clinician, recently-approved antibiotics may be too-poorly differentiated to justify their broad use and inordinate cost relative to those already existing. Perhaps we in the antibacterial drug development field must change our way of thinking if we are to survive and thrive. Rather than reflexively developing new β-lactam-β-lactamase inhibitor combinations for every new enzyme that evades our current inhibitors, we should focus discovery and development efforts on agents that revolutionize how we potentiate antibiotics. To this end, there has been renewed interest in phage therapies, virulence inhibitors, bacterial growth rate modulators, monoclonal antibodies, and other approaches to augment antibiotic effects. Herein, we suggest that the unmet medical need is less about adding poorly-differentiated antibiotics to our armamentarium and more about the need for innovation in how we augment antibiotic regimen effects.

## 1. The Broken Antibiotic Marketplace

Antibacterial resistance is a well-documented serious and global threat. Drug-resistant organisms infect millions of people and kill tens of thousands every year in the United States alone [1,2]. Despite the urgent need for innovation to meet the antibacterial-resistance challenge, the antibacterial drug pipeline can be viewed as somewhat unhealthy. The majority of newly-approved antibacterial agents or those in late-phase clinical development are retreads from an earlier era (e.g., fosfomycin and sulopenem), marginally improved versions of old drugs (e.g., omadacycline, eravacycline, plazomicin, ceftolozane, tedizolid, and glycopeptide-derivatives), broad-spectrum β-lactamase inhibitors (e.g., avibactam, relebactam, and vaborbactam), potentially resistance-prone single-target agents (e.g., fosfomycin and colistin derivatives), or are from a class new to human use, but are too poorly differentiated from generic agents (e.g., lefamulin). The basis for the lack of innovation may be due to the risk associated with successful discovery, development, and commercialization. Investors may have a low appetite to fund an innovative program in an environment where the lack of commercial success is the norm.

The consequence of the current environment is that a malaise has fallen over the antibacterial drug development community, and this malaise is coupled with the perception that the marketplace is broken [3,4,5]. Antibacterial drug development programs require hundreds of millions of dollars in investment and fail to generate enough revenue to recoup the initial investment let alone being able to use revenue to reinvest in any future drug development programs. It is now common for companies with market-cap values in excess of USD 0.5 to 1 billion prior to product launch to sell for pennies on the dollar a few years after market introduction (e.g., Achaogen, Tetraphase).

For instance, it is estimated that Achaogen spent approximately USD 1 billion to develop plazomicin, a total that includes USD 124.4 million government funding support from the Biomedical Advanced Research and Development Authority (BARDA) [6,7]. Achaogen commercially launched plazomicin in July 2018 and accrued a mere USD 800,000 in 2018 sales [8] before filing for bankruptcy protection in April 2019 [9]. A few months later, Cipla USA Inc. acquired near worldwide rights for cash and considerations in excess of USD 15 million [10]. Thus, an investment of USD 1 billion resulted in a value of USD 15 million! The opportunity to recoup the above-described investment further decreased when Cipla Europe NV recently withdrew their marketing authorisation application for plazomicin based on pharmacoeconomic considerations [11,12].

While plazomicin may represent the most notable of the recent antibacterial commercial failures, there have been numerous other agents that have struggled to generate significant revenue, including dalbavancin (Allergan), delafloxacin (Melinta), eravacycline (Tetraphase), meropenem-vaborbactam (Melinta), oritavancin (Melinta), and tedizolid (Merck). A similar fate could await soon-to-be commercialized antibacterial agents (e.g., fosfomycin, lefamulin [Nabriva], omadacycline [Paratek], and several β-lactam-β-lactamase inhibitor combinations).

In response to the marginally innovative antibacterial pipeline and poor commercial performance of antibacterial agents, the government and other interested groups (e.g., Gates Foundation, Wellcome Trust) are incentivizing antibacterial development. They broadly support two types of incentives, “push-incentives” and “pull-incentives”. Push-incentives are designed to decrease the cost of antibacterial development, while pull-incentives facilitate higher returns on investment. Predominate among sources that provide push-incentives are BARDA and Combating Antibiotic Resistant Bacteria Biopharmaceutical Accelerator (CARB-X), which together support more than 70 projects [13,14]. Pull-incentives aid in decreasing the time duration of regulatory reviews, patent extensions, and premium pricing strategies. (i.e., Developing an Innovative Strategy for Antimicrobial Resistant Microorganisms (DISARM) Act).

Regardless of the efforts to incentivize antibacterial drug development, the fundamental hurdles to commercializing new antibiotics remain. Given this circumstance, perhaps it is time to reassess our thinking with regard to the root cause for the antibacterial industry’s failure to thrive. Specifically, is the perception of a broken antibacterial marketplace correct?

## 2. The Carbapenem-Resistant Enterobacteriaceae Threat

Let us consider two agents, plazomicin and meropenem-vaborbactam, developed to treat antibiotic-resistant bacteria. Plazomicin was reviewed under the Limited Pathway for Antibacterial and Antifungal Drugs (LPAD), while meropenem-vaborbactam was approved prior to LPAD’s implementation. The LPAD mechanism allows the Food and Drug Administration (FDA) to approve antibacterial and antifungal drugs to treat serious and life-threatening infections in a limited patient population with a medical unmet need [15]. Using this regulatory pathway, pharmaceutical companies accept narrow product use labels that include a benefit–risk assessment and government pre-review of marketing materials in exchange for FDA approval based upon limited clinical data.

Plazomicin and meropenem-vaborbactam were each developed to combat carbapenem-resistant Enterobacteriaceae (CRE). The United States Centers for Disease Control and Prevention (CDC) has categorized CRE as an *“urgent threat”* in its 2013 and 2019 Antibiotic Resistance Threats in the United States reports [1,16]. Mobile gene elements that encode for β-lactamase enzymes are the predominant mechanism for bacterial resistance to carbapenem-class antimicrobial agents among Enterobacteriaceae.

*Klebsiella pneumoniae* is the dominant CRE species, comprising 71.1% of all such isolates collected from 2013 to 2016 across global geographical regions [17]. Approximately 2.9% Enterobacteriaceae isolates from North America were reported to be carbapenem-resistant, most often due to *K. pneumoniae* carbapenemase (KPC) expression (54.2%) [17]. While there is great variability in CRE prevalence worldwide, it may be decreasing in North America. After reaching a peak in 2013 of 12%, CRE prevalence decreased to 2.9% by 2016 [17]. Although the prevalence of CRE in North America is currently low, there remain non-carbapenem generic therapeutic alternatives for many infected patients. For example, 63.2, 96.7, 78.3, and 83.3% of CRE, based on isolates collected from United States hospitals from 2016 to 2018, were categorized as susceptible to minocycline, tigecycline, amikacin, and colistin, respectively [18]. Moreover, while only 18.4 and 48.7% of this collection of CRE isolates were susceptible to levofloxacin and gentamicin, respectively [18], these agents are still considered useful for the treatment of patients with urinary tract infections, since such infections are less invasive and these agents concentrate in urine.

To respond to the multi-drug resistant CRE threat, four agents, ceftazidime-avibactam, imipenem-relebactam, meropenem-vaborbactam, and plazomicin, recently received FDA approval, and no less than 14 are currently in development (Table 1). These include representatives from at least five antibacterial classes, including aminoglycoside, monobactam, and inhibitors of β-lactamase, phosphoenolpyruvate synthetase, and LpxC. In the CDC’s 2019 Antibiotic Resistance Threats Report, CRE accounted for 13,100 cases in 2017 [1]. Assuming a USD 1000 per day antibacterial cost, 14-day treatment duration, and no generic antibacterial agent is active, the CRE market size estimate is approximately USD 183 million per year. If the USD 183 million was evenly distributed among the recently FDA-approved CRE antibacterial agents (i.e., ceftazidime-avibactam, imipenem-relebactam, meropenem-vaborbactam, and plazomicin), the resultant revenue would not support the development investment for these three agents, let alone that of the 14 additional agents currently in development.

It is important to recognize that other than possessing CRE activity, the vast majority of these new and emerging therapies are not differentiated from generic agents and cost much more. Maybe the lesson to be learned from the plazomicin and meropenem-vaborbactam commercial failures is not that the market for new antibacterial agents is broken, but rather that these products did not provide sufficient value (from either an efficacy and/or safety perspective) in the collective eyes of clinicians. To build a value proposition sufficient for commercial success, antibacterial discovery and development efforts should focus on agents that revolutionize how we treat infected patients.

## 3. Phage Therapies, Virulence Inhibitors, Bacterial Growth Rate Modulators, and Monoclonal Antibodies

There has been renewed interest in therapeutic and adjuvant alternatives to small molecule antibacterial agents. Among these are phage therapies [19,20], virulence inhibitors [19,21], bacterial growth rate modulators [22,23,24], monoclonal antibodies [25,26,27], and other approaches for meeting the challenge of antibiotic resistance. Of the 37 programs in the CARB-X portfolio for antibacterial treatment, over 48% are alternatives to traditional small molecule antibacterial agents [28]. Many products in various stages of development, from hit-to-lead to preclinical and clinical, that utilize these novel approaches are described in Table 2 [25,26,27,28,29,30,31,32,33,34,35,36,37,38].

For example, monoclonal antibodies have revolutionized cancer chemotherapy [39,40] and have the potential to do the same for the treatment and prevention of infectious diseases [25,26,27]. Even before their much-publicized role in the diagnosis, prevention, and treatment of COVID-19 [41,42,43,44], several infectious disease-focused antibody programs entered clinical testing [25,26,27,29]. To date, three monoclonal antibodies, bezlotoxumab (Merck), obiltoxaximab (Elusys Therapeutics), and raxibacumab (GlaxoSmithKline), have received regulatory agency-approval [45,46,47]. In the case of bezlotoxumab, an innovative monoclonal antibody adjuvant to standard antibacterial therapy which neutralizes *Clostridium difficile* toxin B, the recurrence of *C. difficile* infection, a common sequela to antibacterial therapy, was prevented [45,48].

One monoclonal antibody entering investigational new drug (IND) enabling studies is BB200 (Bravos BioSciences). BB200 targets the lipopolysaccharide (LPS) O-antigen, which is an abundant and accessible antigen on the outer cell wall of Gram-negative bacilli [32]. While O-antigens are highly variable among Enterobacteriaceae, there are four dominant serotypes among *K. pneumoniae* isolates [49]. BB200 targets the O2V (gal-III) serotype and provides activity against greater than 90% of KPC-producing *K. pneumoniae* isolates [32]. BB200’s mechanism of action is three-fold; a direct bactericidal effect mediated by complement binding, opsonophagocytic activity, and LPS neutralization [32]. Dominant among these is the direct acting bactericidal effect, which requires BB200 binding to the bacteria cell surface and plasma complement to cause cell lysis.

**Figure 1:**
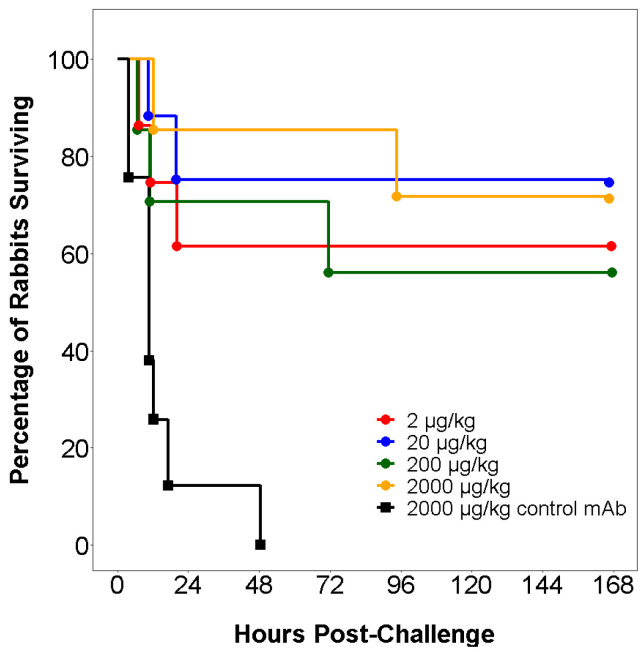
Time-course of animal survival in a lethal rabbit bacteremia model, where animal cohorts (8 per group) received either an intravenous inactive control antibody or one of four BB200 dose levels and then 24 h later challenged with a 100% lethal inoculum of *K. pneumoniae* ST258 (5 × 10^9^ CFU/kg) adapted from reference [32].

BB200 has demonstrated activity against KPC-producing *K. pneumoniae* in a variety of in vitro and in vivo assays [32]. For example, BB200 was studied in a rabbit bacteremia model where animals were challenged with a lethal inoculum (5 × 10^9^ CFL/kg) of *K. pneumoniae* ST258. This model was considered relevant, as both complement activity and endotoxin susceptibility in rabbits are similar to that of humans. As shown in Figure 1**,** all animals treated with an inactive control antibody died by 48 h, while 60 to 80% animals treated with a single BB200 dose as low as 2 mcg/kg survived [32].

**Figure 2:**
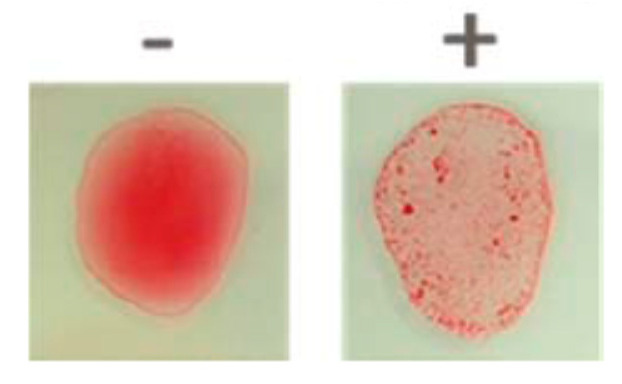
A Bead agglutination assay demonstrating BB200 coupling to colored latex beads (**right panel**) and detecting *K. pneumoniae* ST258; while an inactive control antibody does not (**left panel**).

With the BB200 *proof-of-principle* having been demonstrated across multiple infection models, one can begin to think about the challenges surrounding clinical development. Perhaps the greatest challenge is identifying patients with infection associated *K. pneumoniae* ST250 (serotype O2v). To meet this challenge, the development of a rapid in vitro diagnostic to identify the O2v antigen would be essential. Figure 2 shows a latex bead agglutination assay with BB200 and the detection of *K. pneumoniae* ST250. Completion of a lateral flow assay will allow direct analysis from clinical specimens, including urine, blood, and pulmonary aspirates, and will support the study of BB200 in patients with urinary tract infection, bacteremia, and pneumonia.

The idea of using bacteriophages (“phages”) to combat infections is not new. After they were first discovered by Frederik Twort in 1915 and Felix d’Herelle in 1917, phages were used to treat infections such as dysentery and cholera. Phage therapy began to fall out of favor in the West after World War II due to the advent of antibiotics along with mixed clinical results and production difficulties with phages. However, phage therapies, which were available for a wide variety of indications, continued to be utilized in the former Soviet Union and Poland [20,50,51].

With the need for innovative treatments for patients with infections arising from resistant bacteria, there has been a renewed interest in phage therapy in recent years. Additionally, technological advances, which make phage therapy more viable than in the past, allow for improved characterization and production capabilities [50]. While some phages target multiple species or genera, most are highly specific for a single strain [52]. Single phages are highly predisposed to resistance development and therefore are often combined into multiple phage cocktails or, to achieve synergy, administered in combination with small molecule antimicrobial agents [50,52,53,54,55].

The Eliava Institute of Bacteriophages, Microbiology and Virology in Tbilisi, Georgia, which has been treating patients using phage therapy for nearly 100 years [20,56], is currently using phage therapy to treat infections caused by CRE and other resistant bacteria [56]. The Phage Therapy Center of the Hirszfeld Institute of Immunology and Experimental Therapy in Wroclaw, Poland offers experimental phage therapy to patients infected with certain Gram-negative and -positive bacteria [57]. In addition to the treatment offered by these centers, a number of clinical studies with favorable results using phage therapy have been conducted worldwide in the past decade [50]. Furthermore, some CRISPR-based phages are currently in development to prevent and treat infections. CARB-X announced in June 2020 that it has awarded funding to Eligo Bioscience for the development of CRISPR-based phages to eliminate extended-spectrum β-lactamase (ESBL)-producing and CRE from the microbiome of organ transplant patients before their procedure in order to prevent the onset of infection [58]. Locus Biosciences announced in January 2020 that enrollment for a Phase 1b clinical study of their CRISPR-based phage product LBP-EC01 targeting *Escherichia coli* had begun [38].

While lysins are not phages, lysins are an important part of the phage life cycle. After the phage has replicated inside the host cell, lysins degrade the cell membrane, thereby releasing the newly-synthesized phages [51]. Among the lysins in development, exebacase (formerly known as CF-301) [59] and N-Rephasin SAL200 (tonabacase) [37] have been evaluated in patients. A Phase 2 clinical study of exebacase was completed in 2019 in patients with *Staphylococcus aureus* bacteremia, including endocarditis [59]. In this study, exebacase was given as an adjunct treatment to the standard of care. Patients treated with exebacase demonstrated a significantly higher clinical responder rate in the methicillin-resistant *S. aureus* (MRSA) subgroup at the primary efficacy time point at Day 14 compared to those in the MRSA subgroup treated with antibiotics alone (74.1 vs. 31.3%, *p* = 0.01) [59]. A Phase 3 study evaluating exebacase in patients with *S. aureus* bacteremia including endocarditis is currently being conducted [60].

Phage therapies, virulence inhibitors, bacterial growth rate modulators, and monoclonal antibodies all have the potential to be used in the treatment of infectious diseases either alone or as adjuvants to existing therapies. While rapid in vitro diagnostics are increasing in availability and are critical to identifying patients who might benefit from new technologies, many challenges remain in the design of clinical studies to demonstrate the benefit of such agents. Some questions that need to be asked are the following: Are there target density (bacterial, virulence factors, etc.) thresholds above which the novel agent is rendered ineffective and are some of these novel approaches best suited to be small molecule antibacterial adjuvants? The answers to these questions and others will come in time, and regulatory pathways will likely need to continue to evolve. The moment has come for the antibacterial drug discovery and development community to commit to innovation rather than continue to advance poorly differentiated antibiotics and expect assured commercial success.

## 4. Conclusions

Due to the commercial failure of many recently-approved antibiotics to treat drug-resistant bacteria, many have concluded that the marketplace for new antibiotics is broken. Alternatively, the commercial failure of these new agents may be due to the narrow differentiation with other available agents. This lack of sufficient differentiation, together with the inordinate cost compared to other agents, has made the justification for their use challenging. Our repeated decisions to advance poorly differentiated agents into clinical development suggest that our collective thinking as an antibacterial drug development community has calcified. There is a need to refocus discovery and development efforts on agents that revolutionize how we treat infectious diseases. To this end, there has been renewed interest in phage therapies, virulence inhibitors, bacterial growth rate modulators, monoclonal antibodies, and other approaches to augment antibiotic effects. Such approaches hold the promise to revolutionize how we will use antibacterial agents clinically to care for patients. It is time to move on from the current safe but commercially untenable approach centered on small molecules that provide incremental improvements to existing therapies.

## Figures and Tables

**Table 1:** Agents approved or in development active against CRE.

Approved Agent	Class	Developmental Agent	Class
Ceftazidime-avibactam	β-lactam-β-lactamase inhibitor	Apramycin	Aminoglycoside
Cefiderocol	Siderophore cephalosporin	Arbekacin inhalation	Aminoglycoside
Ciprofloxacin	Fluoroquinolone	BOS-228	Monobactam
Colistin	Polymyxin	Cefepime-AAI101	β-lactam-β-lactamase inhibitor
Doxycycline	Tetracycline	Cefepime-VNRX5133	β-lactam-β-lactamase inhibitor
Imipenem-relebactam	β-lactam-β-lactamase inhibitor	Cefepime-zidebactam	β-lactam-PBP2 inhibitor
Levofloxacin	Fluoroquinolone	Ceftibuten-VNRX7145	β-lactam-β-lactamase inhibitor
Meropenem-vaborbactam	β-lactam-β-lactamase inhibitor	Cefpodoxime-ETX0282	β-lactam- β-lactamase inhibitor
Plazomicin	Aminogycoside	FG-LpxC-UTI	LpxC inhibitor
Tigecycline	Tetracycline	Fosfomycin ^1^	Phosphoenolpyruvate synthetase inhibitor
		KBP-7072	Tetracycline
		Meropenem-nacubactam	β-lactam-β-lactamase inhibitor
		QPX2015-QPX7728	β-lactam-β-lactamase inhibitor
		SPR206	Polymyxin

^1^ IV fosfomycin is currently available in many regions outside the United States.

**Table 2:** Novel investigational therapeutic approaches to treat patients infected with antibiotic-resistant bacteria.

Sponsor	Product	Class/Mechanism of Action	Reference
Amicrobe, Inc.	Amicidin-β	Amicidin	[28]
Antabio SAS	PEi	Pseudomonas elastase inhibitor	[28]
Aridis Pharmaceuticals	Aerumab ^1^, AR-401, Salvecin ^2^	Monoclonal antibodies	[25,26,27,29]
Armata Pharmaceuticals	AP-PA02, AP-SA02	Phage cocktails	[30,31]
AstraZeneca PLC	Suvratoxumab ^3^	Monoclonal antibodies	[25,26,27]
Bravos BioSciences	BB100 ^4^, BB200 ^5^	Monoclonal antibodies	[27,28,32]
Bioharmony Therapeutics	BH01	Lysin	[33]
BioVersys AG	AVATAR-SA	Antivirulent (inhibits AgrA)	[28]
Centauri Therapeutics	ABX01	Dual-acting immunotherapy	[28]
Combioxin SA	CAL02	Antivirulent liposome	[34]
ContraFect Corporation	Exebacase ^6^, Gram-negative lysins	Direct lytic agents	[28]
Eligo Bioscience	EB004	Bacteriophage	[28]
Facile Therapeutics	Ebselen	Anti-toxin	[28]
GangaGen, Inc.	P128	Lysin	[35]
Genentech, Inc.	DSTA4637S	Monoclonal antibody-antibiotic conjugate	[36]
Integrated BioTherapeutics Inc.	IBT-V02	Multivalent toxoid vaccine targeting *Staphylococcus aureus*	[28]
iNtRON Biotechnology, Inc.	N-Rephasin SAL200 ^7^	Lysin	[37]
Locus Biosciences	LBP-EC01	Bacteriophage	[38]
Lytica Therapeutics	StAMPs	Stapled antimicrobial peptides	[28]
Microbiotix, Inc.	*Trans* translational inhibitor	*Trans* translational inhibitor antibiotic	[28]
Microbiotix, Inc.	T3SS inhibitor	T3SS inhibitor, virulence modifier	[28]
MicroPharm Ltd.	PolyCAb	Polyclonal antibody	[27]
Roche	RG7861	Monoclonal antibody	[27]
Seres Therapeutics	SER-155	Microbiome transplant	[28]
Techulon Inc.	PPNA-XPA	Peptide-peptide nucleic acid	[28]
Trellis Bioscience	TRL1068	Monoclonal antibody	[27,28]
Vaxcyte, Inc. ^8^	VAX-A1	Carbohydrate conjugate vaccine targeting Group A *Streptococcus*	[28]
Vaxxilon AG	VXN-319	Multivalent vaccine targeting *K. pneumoniae*	[28]
VaxDyn, S.L.	VXD-003	Monoclonal antibody	[27]
Vedanta Biosciences, Inc.	VE707	Live biotherapeutic	[27]
XBiotech Inc.	514G3	Monoclonal antibody	[25,27]

^1^ Also known as AR-101 and panobacumab. ^2^ Also known as AR-301 and tosatoxumab. ^3^ known as MEDI4893. ^4^ Previously known as ASN-4. ^5^ Previously known as A1102 and ASN-5. ^6^ Previously known as CF-301. ^7^ Also known as tonabacase. ^8^ Previously known as SutroVax, Inc.
